# Exploring the Lived Experiences of Iranian Adolescents Exhibiting Suicidal Behavior and Ideation

**DOI:** 10.1007/s10802-025-01295-0

**Published:** 2025-02-10

**Authors:** Khatereh Arbabi, Christine Jean Yeh, Parvaneh Rahmati Sangkar

**Affiliations:** 1 Counselling and Guidance, Department of Education, National University of Malaysia (UKM), Bangi, Selangor Malaysia; 2 Department of Counselling Psychology, School of Education, University of San Francisco, 2130 Fulton Street, San Francisco, CA 94117 USA; 3 Department of Psychology, Islamic Azad University of Science & Research Branch, Tehran, Iran

**Keywords:** Adolescent suicide, Suicide prevention, Iran

## Abstract

Suicide is a complex phenomenon influenced by various individual and contextual factors that may inform prevention efforts. This research is part of a comprehensive project conducted in the chain clinics of Iris Company (pseudonym) throughout Iran. 1). The objective of the study was to expand the current literature on adolescent suicidality by qualitatively exploring the lived experiences of adolescents with a history of at least one suicide attempt and suicidal ideation through in-depth semi-structured interviews. 2). Participants: The sample included 69 Iranian adolescents (38 females and 31 males) aged 15–19 years from diverse cultural and societal backgrounds across various regions of Iran. 3). Results: Data were analyzed using the Colaizzi method of phenomenological analysis (Praveena & Sasikumar, 2021), resulting in six main themes and 14 sub-themes. The main themes were: (1) overwhelming emotional pain, (2) influence of traditional cultural values, (3) desire for stronger connections, (4) openness to treatment, (5) wish to improve life, and (6) feeling trapped in the present. 4). Conclusions: This study addresses gaps in prior research and aims to advance the understanding of adolescent suicide prevention. The authors emphasize the importance of family and relational contexts in understanding suicide risk among adolescents and highlight the roles of family therapy and peer support in developing prevention and intervention strategies for at-risk youth.

**Supplementary Information:**

The online version contains supplementary material available at 10.1007/s10802-025-01295-0.

The National Institutes of Health define suicide as a deliberate and voluntary act of intentionally ending one’s life (Gusmão et al., 2013). The risk factors and strategies for dealing with and managing suicide vary, reflecting the cultural, psychological, and social context of the society in which it occurs (Kirmayer, 2022; Zarani & Ahmadi, 2021). Research on suicide risk factors has examined various individual and contextual elements, as well as the utility of multi-informant data (Tan et al., 2018; Van Dulmen et al., 2011), focusing on psychological issues, traumatic experiences, family traits, and cultural factors.

## Adolescent Suicide

In recent decades, adolescent suicide has been on the rise, causing widespread concern in schools, families, and communities globally (Costanza et al., 2021; Leigh et al., 2023; Moon et al., 2020). Suicide is now the second leading cause of death among young people (Mokhtari et al., 2019) and the fourth leading cause of death among adolescents aged 15 to 19 (Mars et al., 2019). Quantitative studies have identified numerous factors linked to suicidal behavior, including personality disorders, depression, emotion-focused coping styles, psychological distress, hopelessness, childhood trauma, stressful life events, losses, and substance abuse (Czyz & King, 2015; Glenn et al., 2022; Kleiman et al., 2018; Rahimian Boogar et al., 2014; Rezaei & Ghazanfari, 2016). Recent research further indicates that family conflict and dysfunction play a major role in adolescent suicide risk (Rahmani et al., 2019; Sarkisian et al., 2021; Van Meter et al., 2019). These findings emphasize that suicidal risk in adolescents arises from a combination of individual, interpersonal, familial, and social factors.

### Qualitative and Quantitative Research on Suicide

Qualitative research has revealed the risk factors associated with suicidal ideation and behavior are often connected to cultural identity conflicts and social conditions (Akotia et al., 2019; Chu et al., 2019; Zarani & Ahmadi, 2021), including social rejection, interpersonal issues, social alienation, and an individual’s need for respect and attention (Ghaderzadeh & Piri, 2014; Ryazi & Najafianpour, 2016). Additionally, experiences of interpersonal loss and negative emotions influence these risk factors (Clua-García et al., 2021). Previous studies have identified primary factors contributing to suicide, comprising cognitive, psychological, biological, cultural, social, and environmental variables (Chan et al., 2014; Kuttichira, 2018; Melhem et al., 2007; Nalipay & Ku, 2019).

Quantitative studies consistently demonstrate a significant relationship between a range of psychological, social, and economic factors and suicidal ideation and behavior. Specifically, losses and depression have been identified as positively correlated with suicidal outcomes (Parajuli et al., 2024; Rezaei & Ghazanfari, 2016), as have anxiety and feelings of loneliness. Emotion-focused coping styles and psychological distress are also risk factors, indicating the role of maladaptive coping mechanisms in exacerbating vulnerability (Rahimian Boogar et al., 2014; Rezaei & Ghazanfari, 2016). Furthermore, hopelessness (Rahimian Boogar et al., 2014), economic instability (Rahimian Boogar et al., 2014), and childhood trauma (Rezaei & Ghazanfari, 2016; Tae & Chae, 2021) have been quantitatively linked to higher suicide risk. These studies often rely on structured scales and surveys, such as the Beck Depression Inventory and the Hopelessness Scale, to measure psychological distress and its associations with suicidal behaviors.

In addition to individual factors, interpersonal and contextual elements contribute to suicidal ideation. Social issues, including familial turmoil and conflicts, are key risk factors, underscoring the importance of family dynamics (Parajuli et al., 2024; Rahmani et al., 2019; Ursano et al., 2018). Religious commitments, while often protective, can also exacerbate distress when they create conflicts or feelings of inadequacy (Ghorbanisabagh et al., 2018). Other external stressors, such as exposure to stressful life events (Panadero et al., 2018), substance and alcohol abuse (Peltzer & Pengpid, 2021), and economic hardships, further compound the risk (Rahimian Boogar et al., 2014). These findings underscore the complex nature of suicide risk, highlighting the interaction of individual vulnerabilities with systemic and relational stressors. Quantitative research offers a framework to understand how these factors contribute to suicide attempts.

Complementing this quantitative foundation, qualitative research offers a nuanced understanding of the lived experiences of individuals at risk for suicide, particularly in cultural and social contexts. For example, qualitative studies explore the interplay between identity conflicts, traditional social values, and the subjective experience of feeling trapped, adding depth to the understanding of how these factors manifest in specific populations (Rahimian Boogar et al., 2014; Ghorbanisabagh et al., 2018). By capturing personal narratives and contextual details, qualitative methods highlight the mechanisms underlying quantitative findings, such as how family turmoil (Parajuli et al., 2024; Rahmani et al., 2019) and stressful life events (Panadero et al., 2018) are experienced and navigated by individuals. However, gaps remain, particularly in bridging these approaches to understand the connection of individual vulnerabilities with systemic and cultural influences. The present study seeks to address this gap by integrating qualitative insights into the lived experiences of Iranian adolescents, providing a culturally specific lens to complement the broader quantitative literature.

Protective factors for adolescent suicide include strong family and social support, effective coping skills, and access to mental health care. Positive relationships can foster a sense of belonging and reduce feelings of isolation, which are critical in preventing suicidal ideation (Czyz & King, 2015). Engaging in school and community activities also provides adolescents with a sense of purpose and connection. Open communication, emotional regulation, and problem-solving abilities are key protective factors that help adolescents manage stress and negative emotions (Kleiman et al., 2018).

## Conceptual Frameworks for Adolescent Suicide

Theories of suicide provide important frameworks for understanding the complex factors contributing to suicidal ideation and behavior, particularly during adolescence. However, the applicability of generic suicide theories to adolescents is often limited, as they sometimes don’t include a developmental lens that addresses the unique challenges of this age group. This section integrates key theoretical frameworks while critically reflecting on their relevance to adolescent suicidality, particularly in the context of family and interpersonal relationships.

The Interpersonal-Psychological Theory of Suicide (IPTS) posits that suicidal behavior arises from the interaction of disrupted belongingness, perceived burdensomeness, hopelessness about interpersonal interactions, and acquired capability for suicide (Joiner et al., 2021; Stewart et al., 2017). While quantitative research supports the model’s relevance to adolescents (Al-Dajani & Czyz, 2022; Hill et al., 2019; Opperman et al., 2015), qualitative studies reveal how these constructs manifest in diverse social and cultural contexts. For example, family relationships are critical in fostering a sense of belonging and reducing perceived burdensomeness, which are protective factors against suicide (Opperman et al., 2015). Strengthening these connections, particularly within family and school environments, is a vital preventative measure (Cero & Sifers, 2013; Wong & Maffini, 2011). Despite its empirical support, IPTS may lack a developmental focus and does not fully address how attachment disruptions and family dynamics, unique to adolescents, influence these constructs, signaling a need for more research integrating attachment-based perspectives.

The Integrated Motivational-Volitional (IMV) Model conceptualizes the progression from suicidal ideation to behavior through pre-motivational, motivational, and volitional phases (O’Connor & Kirtley, 2018). Moderators such as perceived burdensomeness, thwarted belongingness, and entrapment provide insight into the pathways linking stress and suicidal ideation. Adolescents, who are particularly vulnerable to peer-related defeat and social stressors, may experience intensified feelings of entrapment, increasing their risk for suicidal behaviors. Although the IMV model has gained empirical support for its applicability to various populations, there is limited research addressing its developmental and family-focused dimensions in adolescents, particularly how family dynamics and attachment ruptures contribute to entrapment and volitional behavior. Future research could explore these connections, offering a more comprehensive understanding of adolescent-specific pathways to suicidality.

The Three-Step Theory (3ST) focuses on the progression from suicidal ideation to attempts, emphasizing psychological pain and hopelessness as precursors to ideation and connectedness as a protective factor (Klonsky et al., 2021). For adolescents, connectedness—particularly parental and social—has been identified as crucial in mitigating suicidal desire (Arango et al., 2019). However, this broad approach to psychological pain and connectedness would benefit from a nuanced examination of developmental factors and the role of family dynamics, including attachment ruptures, in moderating these relationships.

To address the developmental and family-focused gaps in generic suicide theories, family-centered frameworks like Attachment-Based Family Therapy (ABFT) provide valuable insights. ABFT emphasizes repairing attachment ruptures and fostering family cohesion to address adolescent suicidality (Diamond et al., 2021). Similarly, Dialectical Behavior Therapy for Adolescents (DBT-A) highlights how emotional dysregulation and a lack of behavioral skills within the family environment contribute to suicidal behaviors (Miller et al., 2006). Evidence suggests that DBT-A effectively reduces self-harm and suicidal ideation in adolescents with repetitive self-harming behaviors (Mehlum et al., 2014). Additionally, Integrated/Family Cognitive Behavior Therapy (I-CBT) highlights the necessity of parental involvement in emotion regulation, equipping parents with skills to support their adolescents and prevent harmful behaviors (Esposito-Smythers et al., 2019).

These family-based approaches underscore the importance of relational and attachment-focused interventions, yet theories addressing the link between adolescent development and family dynamics in suicidality remain limited. This study provides qualitative insights into how family relationships and cultural contexts influence suicidal ideation, complementing quantitative research and informing culturally sensitive, family-centered prevention strategies.

## International Research on Adolescent Suicide

Goldston et al. (2008) highlighted the critical role of cultural and contextual factors in adolescent suicidal behaviors, noting variations in triggers, protective factors, and help-seeking patterns across diverse groups, including African American, American Indian and Alaska Native, Asian American and Pacific Islander, and Latine youth. They explored acculturative stress, cultural protective factors, and religious influences. The authors also emphasized the potential of culturally sensitive interventions and family dynamics in reducing suicidality, while acknowledging the barriers posed by stigma and cultural distrust. The authors advocate for community-centered prevention approaches and research into culturally tailored strategies.

Similarly, Berg et al. (2014) examined suicidal behaviors in South African adolescents, noting high levels of suicidality. Based on data from 214 at-risk adolescents in the Western Cape Province, the study identified substance abuse, negative emotions, impaired problem-solving, family conflicts, relationship challenges, and socioeconomic stressors as key risk factors. Using the Conservation of Resources (COR) theory, they emphasized building adolescent resources to mitigate these stressors and advocated for targeted interventions addressing the unique psychosocial realities of South African youth.

Grimmond et al. (2019) reviewed 27 qualitative studies, which mapped out key themes related to suicide risk, recovery, and prevention. Their analysis identified triggers such as family and peer influences, cultural stressors, and emotional turmoil, alongside factors promoting recovery, including interpersonal connections and cultural support systems. The authors emphasized the importance of institutional treatment and community-based intervention strategies. By illustrating the complex relationship between cultural and individual influences, the review reinforced the need for culturally grounded and family-centered interventions.

Balaji et al. (2023) examined the experiences of 47 individuals aged 15 to 29 in India who had attempted suicide, identifying background vulnerabilities, psychological distress, and intervening triggers as key factors. Societal norms and interpersonal stressors, especially for women, were significant contributors. The study advocates for life skills programs, targeted interventions for high-risk groups, and involving families in prevention strategies, highlighting the critical role of societal and familial influences on adolescent suicidality.

From a clinician’s perspective, Simes et al. (2024) explored youth suicide intervention through focus groups with 28 clinicians, emphasizing relational and attachment-informed approaches, flexible care for complex family dynamics, and nuanced risk management strategies. The authors highlighted the need for systemic support and better integration of administrative protocols, reinforcing the role of family systems in effective suicide prevention.

This discussion synthesizes evidence on the influence of family relationships and cultural stressors, addressing gaps identified in the present study. It underscores the value of culturally informed, family-centered prevention strategies to improve outcomes for at-risk adolescents.

## Suicide Research in Iran

There has been growing attention to adolescent suicide risk factors in Iran. Mokhtari et al. (2019) conducted an epidemiological study of 6,720 Iranian youth aged 10–19 over six years (2011–2016) and found an increase in suicide attempts and fatalities. Family conflict was a significant contributor, with drug or medication overdose being the most common method. Other risk factors included being male and living in rural areas. Similarly, Ziaei et al. (2017) investigated suicidal ideation among 1,517 high school students in Iran, finding 4.1% had suicidal thoughts and 20.6% reported being bullied in the past month. Cigarette smoking, interest in alcohol and drugs, and a history of sexual abuse also predicted suicidal ideation.

Expanding on this, Bazrafshan et al. (2015) performed a qualitative grounded theory study of 16 adolescents (aged 13 to 19), all of whom had attempted suicide. The study revealed that their primary motivation for attempting suicide was to escape painful psychological conditions. A follow-up study by Bazrafshan et al. (2016) further explored these motivations through interviews with adolescents hospitalized for suicidal drug overdoses, identifying individual, familial, and social factors as key contributors to their suicide attempts.

## The Current Study

Adolescence, particularly between the ages of 15 and 19, is marked by significant physical and psychological changes that increase vulnerability to suicidal behaviors (Badarch et al., 2022; Rajhvajn Bulat et al., 2023). While extensive research has identified risk factors and mental health disorders linked to suicide, most studies have been quantitative, reflecting the complexities of the phenomenon. Consequently, there has been limited qualitative exploration of how suicidal thoughts manifest in adolescents. Suicide does not occur in isolation but is shaped by broader cultural and social frameworks that influence individual experiences. Addressing this gap requires a deeper understanding of the emotions and lived experiences contributing to suicidal ideation and behavior in teenagers.

To address this gap, the present study employs a qualitative phenomenological approach to explore the lived experiences of Iranian adolescents who exhibit suicidal behaviors and ideation. This method enables participants to share their feelings and insights, building on prior quantitative research on suicide (e.g., Hjelmeland & Knizek, 2010). Phenomenological inquiry offers a nuanced exploration of how adolescents experience and interpret their mental health struggles, making it well-suited for understanding the formation of suicidal thoughts.

By focusing on Iranian adolescents with a history of suicide attempts and ongoing suicidal ideation, this study aims to examine the psychological, emotional, and interpersonal dimensions of their experiences. Through this lens, the research seeks to uncover how these adolescents perceive and make meaning of the suicidal process, providing valuable insights that could inform future suicide prevention strategies. Understanding these complexities can help predict the development of suicidal thoughts and improve intervention efforts for at-risk youth.

## Method

The phenomenological approach was selected as the most suitable qualitative method for exploring first-hand experiences of suicide (Barekatain et al., 2013; Wang et al., 2016). Specifically, this study employs Colaizzi’s descriptive phenomenology, chosen from among various phenomenological approaches (Edward & Welch, 2011; Praveena & Sasikumar, 2021). This method allows the researchers to acknowledge and interrogate biases, prejudices, and preconceived beliefs about adolescent suicide, facilitating a deeper understanding of the phenomenon and the participants’ perceptual experiences.

### Participants

Participants for this study were recruited through convenience sampling focusing on adolescents aged 15 to 19 who were receiving treatment from licensed mental health professionals at Iris psychotherapy clinics across provincial centers in Iran. These clinics, staffed by psychiatrists, social workers, psychologists, counselors, and psychometricians, provided a supportive setting for the study. The initial pool included 179 adolescents (male and female) within the specified age range, all with a history of at least one suicide attempt and current suicidal ideation. To account for the cultural and social influences on suicidal thoughts (Akotia et al., 2019; Chu et al., 2019; Rezaei & Ghazanfari, 2016; Zarani & Ahmadi, 2021), participants were selected from diverse regions of Iran to ensure broad representation.

Next, a seminar focused on coping skills was offered to the 179 adolescents, and volunteers were invited to participate in the study. Of the 179, 92 showed interest, and 81 met the inclusion criteria. We interviewed participants in the order of who responded to us and met criteria. The interviewers continued to interview and started to sense that they had reached saturation after about 30 interviews. However, they continued to interview all 69 participants to ensure they heard their stories and allowed for a diverse sample. Although larger than typical qualitative studies, this sample size does align with previous phenomenological qualitative research in education (Hennink & Kaiser, 2022).

Among the 69 participants in the study (mean age = 15.8, SD = 1.73), 38 (55%) identified as female and 31 (45%) as male. Their educational levels were distributed as follows: 26 participants (38%) were in ninth grade, 17 (24%) in 10th grade, 14 (20%) in 11th grade, 9 (14%) in 12th grade, and 3 (4%) were university students. Socioeconomic status was assessed using a validated questionnaire that considered factors such as parental education, occupation, and household income. Of the participants, 11 (15.94%) were classified as having a good economic and social status, 43 (62.32%) were in the average range, and 15 (21.74%) were categorized at the lower end of the socioeconomic scale.

### Procedure

The study received ethical approval from the first author’s institution. Informed consent, written in Persian, was obtained from each participant or, in the case of minors, from a parent or guardian, following ethical guidelines for parental consent and child assent. Parents were provided with detailed information about the study and were required to give written consent, while adolescents gave their assent before participating. Participants were assured of confidentiality and informed of their right to withdraw from the study at any time. The sensitive nature and objectives of the research were carefully explained. Once consent was secured, private in-person interviews were scheduled at times convenient for the participants. In this qualitative study with suicidal adolescents, attrition—defined as participant dropout or withdrawal—was identified as a potential factor affecting the depth, breadth, and representativeness of the data collected.

### Researcher Reflexivity

The research team consisted of four individuals, three of whom are authors. The first author is an Iranian, female, mental health therapist with a Ph.D. in Clinical Psychology and 13 years of experience as a therapist. She has specialized in acute cases, particularly adolescent suicide, for five years. The third author is an Iranian, female, mental health therapist with a Master’s Degree in Clinical Psychology and seven years of clinical experience, focused on working with individuals who have attempted suicide. The two of them conducted all of the in-depth semi-structured interviews in Persian and translated and coded the data.

The second author, a second-generation Taiwanese-American female professor of Counseling Psychology in the San Francisco Bay Area, served as the auditor of the research. She has a Ph.D. in Counseling Psychology with 20 years of experience working with at-risk youth. She has also been a collaborator for several years on research projects focused on Iranian adolescent mental health. The fourth team member (not an author) is an Iranian female administrator who holds a Bachelor’s degree and was tasked with coordinating necessary activities with clinic officials and patients. None of these four team members had prior familiarity with the patients.

### The Interview Process

To explore participants’ lived experiences with suicidality, researchers began each interview by sharing a story of someone who attempted suicide to encourage openness, a method previously used to model vulnerability (Talebi et al., 2018). Participants were then invited to share and explain their experiences. The main interview questions were based on prior literature (Khatami & Khodabakhshi-Koolaee, 2021; Shamsaei et al., 2020), with the initial question asking what personal experiences led to suicidal thoughts. Subsequent questions, tailored to participants’ responses, explored reactions from close ones, thoughts and feelings before the attempt, overall outlook on life, and the personal significance of life and relationships. Probing questions were used to deepen understanding. Interviews, lasting 40 to 60 min depending on individual circumstances, were recorded with participants’ consent, transcribed, and analyzed. Following transcription and coding, subsequent interviews were conducted to refine and adjust the structured questions, ensuring a deeper exploration of the themes (Yeh et al., 2024).

### Coding and Data Analysis

We used the Colaizzi Method of phenomenological data analysis which has different stages that frame the coding and analytic process (Praveena & Sasikumar, 2021). First the first and third authors of the research team transcribed the text of each interview. This was read multiple times to gain an overall understanding of its meaning. The research team (first and third authors) wrote down notes of important phrases and sentences related to individuals’ experiences with suicide. Key ideas and meanings were then extracted from the transcribed texts. Next, the research team used the notes and ideas to begin to formulate participant meanings, where the concept of important phrases was explained in detail and quotes were gathered. These researchers began to formulate meanings by reading and discussing key ideas and passages from the transcripts to address commonalities and differences. All formulated meanings were grouped into distinct thematic clusters to help organize participants’ experiences.

The authors combined and integrated these thematic clusters to form separate thematic structures. For example, they used reductions to eliminate redundant and exaggerated descriptions that weakened the comprehensive description. They also created new themes to help explain phenomena that were emerging but not in a specific thematic cluster. The researchers also slowly developed themes regarding the participants’ lived experiences. This involved removing some ambiguous and double-sided structures that compromised the comprehensive description. The researchers continued to discuss the codes and ideas and began to develop themes and sub-categories (Yeh et al., 2021).

### Validity and Reliability of Data

To ensure credibility, transcripts were provided to 37 of the 69 participants for review, enabling them to identify and correct any misinterpretations, omissions, or errors. Data analysis began with the revised transcripts, which were reviewed multiple times to extract meaningful units. These units were condensed into codes, which were categorized based on conceptual and semantic similarities to ensure internal consistency and clear distinctions between categories.

To enhance confirmability, the researcher reflected on potential biases, assumptions, and preconceptions about the research. For dependability, the transcripts were independently coded by the third author, who acted as an auditor. She compared her findings with the research team’s analyses, provided feedback on themes, and met with the team to discuss revisions, which were incorporated as necessary. This process resulted in 451 initial codes or conceptual statements, analyzed using descriptive phenomenological methods.

Ethical considerations included informing participants about the study’s objectives, obtaining informed consent, securing authorization to record interviews, and ensuring confidentiality through the use of pseudonyms when necessary. Participants were also given the option to withdraw from the study at any time, with their privacy strictly upheld.

## Results

The coding and data analytic process, along with feedback from participants and the auditor resulted in six main themes and 14 sub-categories (see Table 1). The analysis revealed notable patterns in the prevalence of themes across demographic and contextual factors. Themes 1 through 4 were predominantly expressed by girls, with Theme 1 being most common among Kurdish participants, Theme 2 among Lor participants, and Themes 3 and 4 among Fars participants. Socio-economic status played a role, as Themes 1, 3, and 4 were frequently reported by individuals from medium socio-economic backgrounds, while Theme 2 was most prevalent among those from low socio-economic backgrounds. In terms of setting, Themes 1, 3, and 4 were more commonly reported by participants in regional areas, while Theme 2 was more associated with rural settings.

**Table 1:** Summary of themes, sub-categories, and sample quotes

	Theme	Sub-categories	Sample quote
1	Overwhelming Emotional Pain	(a) Feelings of worthlessness (b) Struggle with coping and enduring emotional pain	*“I could no longer cope with my broken state*,* burdened by the weight of sadness that life had thrust upon me. To be honest*,* I am disappointed in myself and feel I lack value.“*(feelings of worthlessness)
2	Influence of Traditional Cultural Values	(a) Feeling powerless, (b) self-criticism and (c) idolizing others	*“Honestly*,* my family has their own rituals and specific habits that I cannot ignore. It is not within my control to desire or not desire something. Decisions are made by someone else*,* and others carry them out. This can make one feel worthless and like life has no meaning and is absurd.”* (self-criticism)
3	Desire for stronger connections	(a) sharing feelings and struggles with others and (b) willingness to be vulnerable	*“Every time I discuss my feelings*,* conflicts*,* and confrontations with others*,* they tend to distance themselves from me rather than getting closer. Eventually*,* I catch myself saying*,* “Wow*,* you really bring a lot of negative energy to people.“*(sharing feelings and struggles with others)
4	Openness to treatment	(a) interest in seeing experiences from a different perspective and (b) willingness to accept support	*“I don’t know*,* maybe I scrutinize the issues of my life so much that I don’t understand the realities and can’t find a solution for them. From your perspective*,* how do you see my life story? What would you do if you were in my place?“*(interest in seeing experiences from a different perspective)
5	Wish to Improve Life	(a) desire for different types of entertainment and (b) curiosity about escaping their current situation	*“Despite being judged by many*,* I am determined to figure out how to alter the direction of my life and stay optimistic about my future advancement.”* (curiosity about escaping their current situation)
6	Feeling trapped in the present	(a) unresolved past issues, (b) risky behaviors as a way to feel good in the present, and (c) lack of a plan for the future	*“Maybe it’s not right to say it*,* but drinking alcoholic beverages really lightens me up and at least for a few hours*,* it gives me a sense of calmness“(* risky behaviors as a way to feel good in the present)

In contrast, Themes 5 and 6 were more frequently expressed by boys, with both themes predominantly identified among Fars participants from medium socio-economic backgrounds in regional settings. These patterns highlight gendered and ethnic differences in the expression of themes, as well as variations linked to socio-economic and geographical contexts, underscoring the importance of considering diverse participant characteristics when interpreting the findings.

### Theme 1: Overwhelming Emotional Pain

As participants shared the reasons for their suicide attempts, most of them described *an overwhelming emotional pain* that was all-consuming all of the time. The sub-categories for this theme include (a) feelings of worthlessness and (b) struggle with coping and enduring emotional pain. Specifically, all of the participants described feelings of worthlessness, inadequacy, and being unimportant. For example, Marjan, an 18-year-old participant female stated:

“I could no longer cope with my broken state, burdened by the weight of sadness that life had thrust upon me. To be honest, I am disappointed in myself and feel I lack value.”

These *feelings of worthlessness* contributed to the emotional pain participants were experiencing. Moreover, participants described not being able to find an effective way to *cope with*,* or endure*,* the emotional pain* they were experiencing. They expressed that all they wanted was to not feel this pain and to escape it. In fact, no matter how much they tried, the participants struggled with managing it. Liana, a 15-year-old female participant shared: *“Everything was so rough for me*,* I couldn’t think straight. I cried a lot*,* but nothing worked for me*,* so I just took a pill to put me to sleep to calm down and forget about it.”*

All of the participants admitted that their suicide attempt(s) were linked to their overwhelming emotional pain, feelings of worthlessness, and struggle with coping and enduring this emotional pain.

### Theme 2: Influence of Traditional Cultural Values

Most of the participants expressed the *influence of traditional cultural values* that impacted their ability to cope with the emotional pain. Participants in this study described themselves as bound by a strict upbringing which emphasized traditional cultural values and practices that had been inculcated in them through familial and traditional means. The sub-categories for this theme were (a) feeling powerless and (b) self-criticism (c) idolizing others. For example, most of the 69 participants shared *feeling powerless* in their capacity to change anything in their lives. They felt that their traditional households contributed to a predetermined situation and their daily experience in their upbringing. For example, Yasaman, a 16-year-old participant female explained:

“Honestly, my family has their own rituals and specific habits that I cannot ignore. It is not within my control to desire or not desire something. Decisions are made by someone else, and others carry them out. This can make one feel worthless and like life has no meaning and is absurd.”

Another sub-category of influence from *traditional cultural values* was self-criticism. Participants shared that their upbringing contributed to strong feelings of self-criticism, with feelings of worthlessness and emotional pain stemming partly from family influences. When experiencing emotional pain, they would analyze the reasons, focusing on their limitations, which reinforced their sense of worthlessness. Many admitted that their judgmental mindset and self-criticism had hindered constructive activities. For example, Arghavan, a 16-year-old female participant said,

“My late father was extremely strict. If we didn’t do things exactly as he wanted, he would scold and reprimand us. In all honesty, I don’t recall him ever praising me. For some reason, I often find myself criticizing myself, as though an internal voice resembling my father’s is constantly monitoring and chastising me. This inner voice instills fear of the potential repercussions every time I wish to take action.”

On the other hand, most participants had an idolized figure in their lives whom they wanted to emulate. They tried to think, dress, and live like this person, perceiving them to be flawless and free from any mistakes or wrongdoings. However, at the slightest sign of imperfection or negligence from this idol, their idea for their life would crumble, leaving them feeling worthless once again. For example, Khashayar, a 17-year-old participant male, said,

“My room was covered in his pictures, and seeing his style made me feel so good. It may be hard to believe, but I even modeled my hair after his and adopted his clothing choices and colour preferences. However, when he became embroiled in a scandal and gossip began circulating about him, my entire world felt like it crumbled in just a few days. I don’t know how much these critics consider the impact of their words on artists, but the aftermath of the scandal left me devastated.”

### Theme 3: Desire for Stronger Connections

Most participants admitted they long for stronger connections, believing it could alleviate their loneliness and improve their depressed mood. Despite efforts to gain attention and support, they were largely unsuccessful. The sub-categories for this theme were (a) *sharing feelings and struggles with others* and (b) *willingness to be vulnerable*. Many shared their feelings in hopes of forming connections, but this often led to disappointment, loneliness, and lack of motivation, as others criticized them for being negative and low-energy. Tamara, an 18-year-old female participant said, *“Every time I discuss my feelings*,* conflicts*,* and confrontations with others*,* they tend to distance themselves from me rather than getting closer. Eventually*,* I catch myself saying*,* “Wow*,* you really bring a lot of negative energy to people.”*

Most of the participants also tried to be *vulnerable in front of others* because they thought this could help them find more friends, receive more attention, have more people trust them, and ultimately be more successful in forming strong and meaningful relationships. For example, Ali, an 18-year-old participant male said,

“I believed that opening up about my childhood memories would help strengthen our bond, so I shared some personal stories with him. Initially, I noticed a slight change in his behaviour towards me. However, to my surprise, I later overheard those private discussions from a classmate who harbored negative feelings towards me.”

### Theme 4: Openness to Treatment

Most participants, despite often feeling hopeless and unmotivated, expressed openness to treatment. They wanted therapists to notice details in their discussions to better understand their feelings and experiences, believing this openness could help them find solutions. The sub-categories for this theme were (a) *interest in seeing experiences from a different perspective* and (b) *willingness to accept support*. Many were interested in hearing others’ views on their experiences to understand how they were perceived and how others might act in similar situations. For example Nastaran, an 18-year-old participant female said, *“I don’t know*,* maybe I scrutinize the issues of my life so much that I don’t understand the realities and can’t find a solution for them. From your perspective*,* how do you see my life story? What would you do if you were in my place?”*

Most of the participants were fully prepared to find or even create a new path in their lives. Therefore, they clearly showed their willingness to accept support. For example, Mani, a 17-year-old participant male stated: *“Life can be incredibly challenging*,* especially when you lack a supportive elder figure in this world. I have personally exerted great effort to better my circumstances*,* and perhaps with genuine support from someone*,* I can regain my enthusiasm for the things I once wished for.”*

### Theme 5: Wish to Improve Life

Participants expressed a desire to improve their lives, acknowledging they had reached a point of despair despite their efforts. However, they continued seeking solutions. The sub-categories for this theme are (a) *desire for different types of entertainment* and (b) *curiosity about escaping their current situation*. Most believed that creating new experiences, particularly through changing how they engage with entertainment, could help improve their lives. Shaghayegh, an 18-year-old female participant, expressed:

“I believe that the importance of routine and occasional lack of entertainment shouldn’t be disregarded in a fulfilling life narrative. Perhaps exploring diverse forms of entertainment can offer me a new sense of enjoyment, helping to prevent quick discouragement.”

Most of the participants shared that if they survive, they need to overcome their life stories and create conditions that lead to their growth and progress. For example, Sara, 16-year-old female participant explained: *“Despite being judged by many*,* I am determined to figure out how to alter the direction of my life and stay optimistic about my future advancement.”*

### Theme 6: Feeling Trapped in the Present

Many participants expressed that, because tomorrow is uncertain, they focus on the present to avoid returning to their painful past. When reflecting on the past, they tend to devalue, suppress, and silence it within themselves. Since achieving this mental state was difficult, many turned to risky behaviors, like drug abuse, to briefly experience relief and ecstasy. The sub-categories for this theme were: (a) *unresolved past issues*, (b) *risky behaviors as a way to feel good in the present*, and (c) *lack of a plan for the future*.

Most participants revealed an unresolved part of their past they had never addressed, often blaming themselves for it. This self-reproach led to harmful behavioral patterns, which they believed made them oblivious to the present. For example, Alireza, a 19-year-old participant male said:

“When I was young, my father was too busy talking to his friend that he let go of my hand at the store, and I got lost and cried trying to find him. While looking for my father, I encountered a drug addict who wanted to steal me when he realized I was alone. I was scared and ran into the street, and the sound of the car brakes and people’s yelling brought my father to me. But, not only did he not apologize to me, but he also hit me for running into the street, and he never knew what had happened that day.”

Some participants admitted that in order to create good feelings, they often engaged in dangerous behaviors, which brought them peace, even for a short period of time. For example, Ardavan, a 16-year-old participant male stated: *“Maybe it’s not right to say it*,* but drinking alcoholic beverages really lightens me up and at least for a few hours*,* it gives me a sense of calmness”*.

Most of the participants preferred not to think about the future because they believed it would prevent them from living in the present. Therefore, they had no plans for their future and took all kinds of risks in the present. For example, Shahab, a 15-year-old participant male stated: *" I don’t want to sacrifice my today for a tomorrow that may not even come. Yes*,* I have taken a lot of risks*,* but at least I can say to myself that you have lived*,* even if it hasn’t been very fruitful…”* It is clear from the quotes, that the participants had difficulty reviewing their past and reflecting on unresolved issues. This led to a hyperfocus on the present which included risky behaviors.

## Discussion

A descriptive phenomenological study explored the experiences of 69 Iranian adolescents with suicidal ideation who had previously attempted suicide, identifying six main themes and 14 subthemes. One prominent theme, *Overwhelming Emotional Pain*, was universally acknowledged by participants, revealing the pervasive nature of their distress. Adolescents reported feeling trapped in an endless cycle of suffering, unable to envision relief or alternative solutions, and viewed suicide as the only escape from their anguish. This aligns with the Three-Step Theory of Suicide, which identifies pain and hopelessness as critical risk factors for transitioning from suicidal ideation to attempts (Klonsky et al., 2021; May et al., 2016).

Prior studies have demonstrated that emotional and psychological distress, particularly when compounded by adverse life circumstances, exacerbates feelings of hopelessness and worthlessness, heightening the desire for self-elimination (Balaji et al., 2023; Glenn et al., 2022; Panadero et al., 2018; Wang et al., 2016). These findings contextualize our conceptualization of adolescent suicide as a complex interplay of internalized emotional pain and external life stressors, necessitating multifaceted interventions that address both psychological and environmental factors.

The influence of *Traditional Cultural Values* emerged as another key theme, illustrating how family dynamics and cultural expectations shape adolescents’ thoughts and behaviors. Adolescents described their interactions with family members as pivotal in reinforcing self-destructive tendencies, particularly when cultural norms prioritized reputation, obedience, or idolization of others over emotional well-being. This aligns with theories such as Attachment-Based Family Therapy (Diamond et al., 2021), Family-Based Dialectical Behavior Therapy (Mehlum et al., 2014), and Integrated/Family Cognitive Behavior Therapy (Esposito-Smythers et al., 2019), which emphasize the centrality of family cohesion, emotional support, and attachment repair in mitigating adolescent suicidality. These approaches highlight how unresolved family conflict and rigid cultural expectations can create environments of emotional neglect, thereby contributing to suicidal thoughts and behaviors. Our findings, consistent with prior research (Berg et al., 2014; Goldston et al., 2008; Ghaderzadeh & Piri, 2014; Simes et al., 2024; Zarani & Ahmadi, 2021), deepen this understanding by exploring how cultural values, such as the idolization of role models, can intensify feelings of inadequacy and exacerbate self-destructive behaviors in adolescents. These insights underscore the need to integrate culturally sensitive and family-focused frameworks into conceptualizations of adolescent suicide.

The theme of *Desire for Stronger Connections* was also prominent, shedding light on the fractured identities and relational insecurities experienced by participants. Adolescents frequently described feeling rejected or inadequate, coupled with an intense yearning for meaningful relationships. However, their perceived lack of communication skills or social competence often led to feelings of failure and further isolation. These findings resonate with the Interpersonal-Psychological Theory of Suicide, which posits that hopelessness about interpersonal constructs, such as belongingness and connectedness, can significantly heighten suicide risk (Joiner et al., 2021).

Prior research similarly underscores the protective role of supportive relationships with parents, caregivers, and peers in buffering against suicidality (Cero & Sifers, 2013; Wong & Maffini, 2011; Van Meter et al., 2019; Panadero et al., 2018; Zarani & Ahmadi, 2021). In our conceptualization, the inability to form meaningful connections represents both a risk factor and a critical point of intervention. Strategies that foster interpersonal skills and create opportunities for adolescents to feel valued and supported could be instrumental in reducing their sense of isolation and despair.

The theme of *Openness to Treatment* highlighted participants’ readiness to seek help and their willingness to engage with therapeutic processes. Adolescents expressed an interest in exploring new perspectives, accepting support, and actively working through their challenges.

This theme expands the focus of suicide prevention by emphasizing the importance of understanding adolescents’ needs and desires in treatment, rather than solely addressing their symptoms or risks. Our findings suggest that aligning treatment approaches with adolescents’ openness to change can foster trust, engagement, and long-term recovery. This perspective broadens traditional conceptualizations of suicide, which often frame at-risk individuals primarily as patients requiring disease management, rather than as agents of their own growth and healing.

A notable theme, *Desire to Improve Life*, reflected participants’ strong motivation to create environments that encouraged curiosity, exploration, and personal growth. Adolescents believed that engaging in new activities and cultivating their problem-solving abilities could provide a sense of purpose and direction. This finding ties into developmental theories of adolescence, which highlight the critical role of exploration and self-discovery during this period. Encouraging adolescents to pursue new interests and experiences can help foster resilience and a more positive outlook on life. Our findings emphasize that interventions targeting personal development, creativity, and curiosity may serve as protective factors, offering adolescents alternatives to destructive behaviors and providing pathways toward fulfillment and self-efficacy.

The theme of *Feeling Trapped in the Present* further illustrated the pervasive sense of despair among participants. While some adolescents acknowledged the importance of mindfulness as a coping strategy, others felt overwhelmed by negative experiences that seemed insurmountable. Feelings of being a burden to their families, social inadequacy, and self-disgust were commonly expressed. These findings align with Joiner et al.’s (2021) emphasis on detachment and perceived burdensomeness as key contributors to suicidal tendencies, as well as O’Connor & Kirtley’s (2018) model linking motivational factors and feelings of inadequacy to suicidal ideation. Participants often resorted to avoidance or risky behaviors in attempts to alleviate their distress, but these strategies only deepened their despair and mistrust in life. Conceptually, this theme highlights the critical need for interventions that address adolescents’ perceptions of their present circumstances, fostering hope, resilience, and adaptive coping mechanisms.

In sum, this study provides a nuanced understanding of adolescent suicidality by identifying specific themes that intertwine emotional, relational, cultural, and developmental factors. These findings not only enrich the existing literature but also advance conceptual frameworks of adolescent suicide, emphasizing the importance of culturally informed, family-centered, and developmentally appropriate prevention strategies. The insights from this study highlight opportunities to create more targeted and effective interventions tailored to the unique needs of adolescents.

### Limitations

This study has several limitations that warrant consideration. One notable limitation is the specific focus on suicidal adolescents receiving treatment at a psychology clinic in Iran. Although participants were drawn from various cities and regions, this focus restricts the generalizability of our findings. We selected this clinic to ensure that participants were in treatment and under the supervision of a mental health professional for safety reasons. Additionally, our sample only included adolescents who had previously attempted suicide. Including those with suicidal ideation but no attempts could have offered more comprehensive insights into the factors that distinguish ideation from attempts. Future studies might address this by incorporating adolescents with ideation, as well as gathering multi-informant data, such as interviews with both parents/guardians and children (De Los Reyes et al., 2015).

Another limitation is the use of single, one-time interviews. While these interviews were in-depth and provided valuable insights into the adolescents’ experiences, a longitudinal approach could improve the study. Future research could adopt a repeated-measures model, where participants are interviewed over time to assess how treatment and other factors influence their clinical progress and suicidal thoughts or behaviors. This would allow for a more dynamic understanding of the effects of treatment over time.

## Conclusion and Implications

Our findings contribute significantly to the development of research on adolescent suicide, particularly from a qualitative perspective. The main themes from this study provide actionable insights for researchers and clinicians developing interventions to prevent suicide and promote mental health during adolescence. These findings are especially relevant given the elevated risk of recurrence in individuals with a history of suicide attempts. In fact, researchers estimated the probability of a repeated suicide attempt after an initial attempt to be one out of five individuals (de la Torre-Luque et al., 2023).

The themes of *Openness to Treatment*,* Wish to Improve Life*, and *Feeling Trapped in the Present* underscore the importance of directly engaging with the lived experiences of adolescents at risk. The focus on the present as a coping strategy is particularly relevant for safety planning interventions and aligns with techniques in dialectical behavior therapy for adolescents (DBT-A; Miller et al., 2006; Mehlum et al., 2014). Incorporating mindfulness and distress tolerance strategies, as emphasized in DBT-A, may offer a way to address this theme within a therapeutic context. Clinicians might also consider integrating these insights into structured safety planning tools tailored to adolescent needs.

The emphasis on family relationships in our findings calls for interventions grounded in contemporary models of family therapy. For instance, Attachment-Based Family Therapy (ABFT; Diamond et al., 2021) and Integrated/Family Cognitive Behavior Therapy (I/FCBT; Esposito-Smythers et al., 2019) provide evidence-based frameworks for addressing adolescent suicidality. These approaches focus on strengthening family dynamics, improving attachment relationships, and equipping families with skills to support the adolescent’s mental health. Family-based DBT-A (Mehlum et al., 2014) is another promising avenue, emphasizing collaborative problem-solving and emotional regulation within the family system. Shifting the focus from earlier theories, such as Minuchin’s family systems approach, to these contemporary models aligns with current best practices in the field.

Our findings also have implications for educational programs for parents, schools, and educators. Training educators to create supportive environments that promote both well-being and academic success is critical. Additionally, parent-focused interventions may foster self-efficacy and equip caregivers with the tools to support adolescents navigating suicidal ideation.

Future research should continue exploring these themes across diverse cultural contexts and through quantitative methods such as factor or path analysis to assess the relative impact of each theme on suicidal behaviors. Given the complex and multifaceted nature of adolescent suicidality, a multidisciplinary approach involving psychiatrists, psychotherapists, psychiatric nurses, and counselors is recommended. Group therapy or counseling, informed by the shared experiences highlighted in our study, could also be a valuable addition to treatment programs. By integrating our findings with evidence-based therapeutic approaches and furthering research in this area, we can develop more effective strategies to support adolescents at risk and reduce the rising rates of adolescent suicide.

## Electronic Supplementary Material

Below is the link to the electronic supplementary material.

Supplementary Material 1

## Data Availability

Not applicable.
