# Supplementary material for: Exploring the Lived Experiences of Iranian Adolescents Exhibiting Suicidal Behavior and Ideation
Source: Res Child Adolesc Psychopathol. 2025 Feb 10;53(6):891–904. doi: 10.1007/s10802-025-01295-0 (PMC12137395; doi:10.1007/s10802-025-01295-0)
Supplement: Supplementary file 1 — Supplementary Material 1 [file 10802_2025_1295_MOESM1_ESM.docx]

**Table 2.**

*Abundance of expression of each of the main themes of research*

| **Suicide Attempt History** | **#of participants** | **% of participants** |
| --- | --- | --- |
| No history | 19 | 27.5 |
| 1-3 times | 39 | 56.5 |
| 4-6 times | 8 | 12 |
| More than 6 times | 3 | 4 |
| Total | 69 | 100 |
| **Suicide Method** |  |  |
| Medication for anxiety or depression | 42 | 61 |
| Self-harm | 14 | 20 |
| Stimulant drugs | 9 | 13 |
| Chemical toxins | 2 | 3 |
| Jumping from a height | 1 | 1.5 |
| Hanging | 1 | 1.5 |
| Total | 69 | 100 |
